# Tension Force Estimation of Cable-Stayed Bridges Based on Computer Vision Without the Need for Direct Measurement of Mechanical Parameters of the Cables

**DOI:** 10.3390/s25133910

**Published:** 2025-06-23

**Authors:** German Michel Guzman-Acevedo, Juan A. Quintana-Rodriguez, Guadalupe Esteban Vazquez-Becerra, Luis Alvaro Martinez-Trujano, Francisco J. Carrion-Viramontes, Jorge Garcia-Armenta

**Affiliations:** 1 Department of Vehicle Engineering and Structural Integrity, Mexican Institute of Transportation, San Fandila 76703, Mexico; michel.guzman@imt.mx (G.M.G.-A.); jaquintana@imt.mx (J.A.Q.-R.); amartinez@imt.mx (L.A.M.-T.); carrion@imt.mx (F.J.C.-V.); 2 Department of Earth and Space Sciences, Autonomous University of Sinaloa, Culiacan 80040, Mexico; 3 School of Engineering & Physical Sciences, Institute of Photonics & Quantum Sciences, Heriot-Watt University, Edinburgh EH14 4AS, UK; j.garcia@hw.ac.uk

**Keywords:** computer vision, smartphone, tension cable, SHM, video-images

## Abstract

Commonly, accelerometers are used to determine the tension force in cables through an indirect process; however, it is necessary to know the mechanical parameters of each element, such as mass and length. Typically, obtaining or measuring these parameters is not feasible. Therefore, this research proposed an alternative methodology to indirectly estimate them based on historical information about the so-called classic instruments (accelerometers and hydraulic jack). This case study focused on the Rio Papaloapan Bridge located in Veracruz, Mexico, a structure that has experienced material casting issues due to inadequate heat treatment in some cable top anchor over its lifespan. Thirteen cables from the structure were selected to evaluate the proposed methodology, yielding results within 3.8% of difference compared to direct tension estimation generated by a hydraulic jack. Furthermore, to enhance data collection, this process was complemented using a computer vision methodology. This involved remotely measuring the vibration frequency of cables from high-resolution videos recorded with a smartphone. The non-contact method was validated in a laboratory using a vibrating table, successfully estimating oscillation frequencies from video-recording with a fixed camera. A field test on eight cables of a bridge was also conducted to assess the performance and feasibility of the proposed method. The results demonstrated an RMS Error of approximately 2 mHz and a percentage difference in the tension force estimation below 3% compared to an accelerometer measurement approach. Finally, it was determined that this composed methodology for indirect tension force determination is a viable option when: (1) cables are challenging to access; (2) there is no line of sight between the camera and cables outside the bridge; (3) there is a lack of information about the mechanical parameters of the cables.

## 1. Introduction

The maintenance and monitoring of cable-stayed bridges are crucial for ensuring their adequate operation under the stress of heavy loads, impacting the structure’s lifespan. Cables supporting a bridge commonly encounter two types of problems: a reduction in their area due to corrosion and/or fracture, and wire-strand slippage in their anchorages. The latter failure could be detected by measuring changes in the cable tension force, correlating with variations in the vibration frequency of the cable [1]. Conventional methods for determining cable tension force involve the use of magnetic permeability, hydraulic jacks/load cells, and vibration measurements [2]. Regarding the vibration method, it entails obtaining the vibration frequency of an individual cable and relating this value to the cable tension force by using cable parameters such as the cross-sectional area or the number of wires associated with mass per unit length. The most employed sensor for measuring cable vibrations and estimating tension force is an accelerometer [3,4]. This sensor provides accurate information, a broad range of sampling rates, and a relatively affordable price for most users. Its installation is generally straightforward, involving attaching the device directly to the structural element. However, certain areas of the bridge cables may be restricted or present limited access for coupling vibration sensors and collecting data from various locations. This limitation makes the method inconvenient for determining tension force in most structures, especially in large cable-stayed bridges.

Nevertheless, with the recent increase in resolution in digital cameras and their affordability, this instrument provides an opportunity to measure vibration remotely without the need for physical contact by integrating it with computer vision techniques. Recently, computer vision has been employed more frequently in structural health monitoring to analyze the behavior of structures from a fixed position [5,6,7] or from multiple perspectives by mounting a camera on Unmanned Aerial Vehicles (UAVs) [8,9]. In the case of cable monitoring using a fixed camera, ref. [10] estimates cable tension force through a computer vision approach during the roof erection stages. The differences between the proposed methodology and a load cell reading were within 5.6%. Displacement measurement from multiple targets has also been demonstrated using a vision-based system [11], validated in cable-stayed footbridge trials for deck deformation and cable vibration by comparing results with data obtained using accelerometers as a reference. In [12], a processing approach is proposed for obtaining the full-field modal parameters of cable vibration. This was achieved using an unsupervised machine learning algorithm and video recordings from the cables. The performance of two different digital image techniques (digital image processing and digital image correlation) has been compared for cable tension monitoring against acceleration [13]. Additionally, ref. [14] presents a methodology for monitoring cable tension force using a moving handheld camera. The camera displacement was determined by tracking specific points on the deck and pylon of a bridge. The maximum variation was ±6.69% of the mean tension due to the environmental conditions during the real-time estimation. Up to this point, the listed methodologies have been collecting data outside of the structure or under ideal conditions in the laboratory.

Other research has also proposed methods measuring tension force while on the bridge. In [15], an image-processing technique was developed for monitoring continuously stayed-cables bridges, yielding tension differences within 5% of the designed tension force. In this instance, the camera was installed on the bridge pylon, providing a stable but difficult-to-access location. In [16], an approach to correct camera motion based on a stable point outside the bridge was proposed. It utilized a matching template and a subpixel estimation, collecting data from the bridge while being affected by environmental vibrations. Reference [17] introduces a methodology based on handheld data collection using a smartphone. The method was validated with a difference below 5% in an excited pedestrian bridge. The research also identified challenges, including addressing measurement campaigns affected by vehicle loads and the application in long-span cable-stayed bridges. Research [18] proposed an alternative vision-based cable displacement measurement approach that captures information when the camera is fixed within a short range at the side of cable. Reference [19] evaluated corrections in camera motion with an inertial system against a methodology that implements a stable point far from the camera. Validation was performed in the laboratory and on a pedestrian bridge that was artificially excited. The approach proposed employing only a template-matching technique to determine cable displacements and minimize camera motion, relying on a stable point positioned far from the instrument. While it does not completely eliminate full camera motion, the current study offers an assessment to quantify discrepancies resulting from the residual camera motion in terms of frequency.

Regarding the case of tension force estimation using UAVs, ref. [20] proposes a method that reduces UAV displacements by combining relative displacements and frequency differences. Similarly, in [21], a deep learning algorithm is applied to eliminate background elements in images, and a mode decomposition method is used to isolate cable vibration from UAV movements. Both methods address measurement challenges on or outside of the bridge, highlighting a key advantage of the UAV. However, based on the taut string model, knowledge of the mass per unit length, the length, and the flexural rigidity of the cable is still required for tension force determination.

There are alternative approaches to estimate cable tension force; for instance, ref. [22] uses the sag of cables according to the parabolic cable theory, measured with a camera installed outside of the bridge. Tension force is calculated based on a single picture, but precision is enhanced by averaging values from multiple images. Reference [23] proposed a method to solve an inverse problem to determine the cable’s length, mass per unit length, and axial stiffness. The solution was based on two steps: (1) the cable length was estimated using a constant value of the mass per unit length and axial stiffness; (2) the rest of the physical parameters were evaluated considering an adjoint method. On the other hand, there are some studies that implement machine learning algorithms for finding mechanical parameters of the cables, such as constraint and bending stiffness [24,25,26,27,28,29].

The distinctive features of the current approach include the camera placement on the structure, the absence of artificial markers in challenging cable access points, and the elimination of the need for measurements of certain mechanical parameters. Moreover, it is designed to be implemented when the bridge is affected by traffic load. While the existing non-contact methods for tension estimation typically rely on information about the mass, length, and flexural rigidity of each cable, this research proposes an alternative approach that avoids the need for direct measurement or estimation of mechanical parameters. The methodology is complemented by a computer vision technique using a camera installed on a tripod within the bridge structure, recording individual cables affected by vehicle loads. The recorded video is then analyzed in post-processing to estimate the cable tension. This methodology was developed in the context of the Rio Papaloapan Bridge case study in Mexico, where previous measurements using contact methods were conducted.

This paper is organized as follows. In Section 2, the studied structure, known as Rio Papaloapan Bridge, and its characteristics are described. Section 3 outlines the methodology for determining tension force without prior knowledge of the cable’s mass, length, and flexural rigidity, along with the image-processing algorithm used to retrieve the vibration frequency of the bridge. Section 4 and Section 5 detail the laboratory and field testing, respectively. Section 6 presents the obtained results, and finally, Section 7 provides the conclusions of the proposed tension force calculation method.

## 2. Rio Papaloapan Bridge

**Figure 1:**
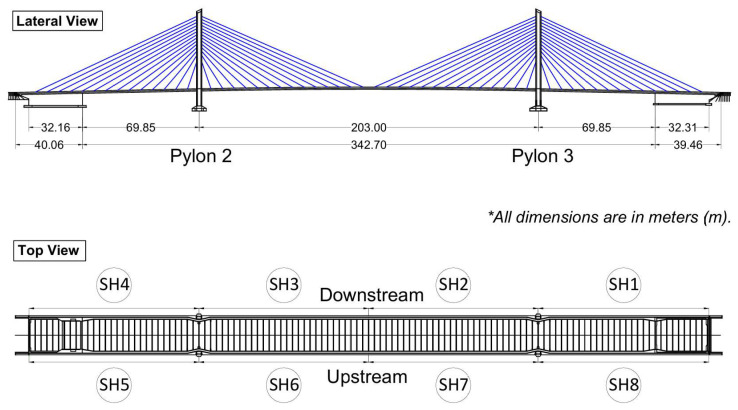
Rio Papaloapan Bridge.

The case study is the cable-stayed Rio Papaloapan Bridge, located in Veracruz, Mexico. It was constructed in 1994, and it features a main span of 203 m and a total length of 407 m. As illustrated in Figure 1, the structure comprises 112 cables distributed among 8 semi-harps, with each semi-harp consisting of 14 cables. Notably, the cables in the first and fourteenth positions are the shortest and largest, respectively.

Table 1 displays the average frequencies of the first to the fifth vibration modes for each cable position. Due to the lower displacements, the shortest cables at the first position generate the highest frequencies, with an average fundamental frequency of 7.5 Hz. Meanwhile, the largest cables at the fourteenth position exhibit an opposite behavior, with an average fundamental frequency of 1.16 Hz. These frequency values were computed based on historical information acquired by accelerometers placed on each cable.

Over the years, the Rio Papaloapan Bridge has faced issues related to casting material due to inadequate heat treatment in certain of the cable top anchors, prompting the implementation of frequent monitoring campaigns. The primary measurements and parameters obtained from the cables include direct tension force and vibration frequency estimation. Direct tension force is measured using hydraulic jacks, while, simultaneously, accelerometers are positioned along the cables of each semi-harp to estimate vibration frequencies. This data forms the basis of the proposed methodology for indirect cable tension estimation.

## 3. Methodology

### 3.1. Tension Force Estimation

The relation between tension force and vibration frequency is established by the following equation, based on the beam and string theories [10]:

(1) $$T=4ml^{2}\frac{f_{n}^{2}}{\gamma_{n}^{2}}-\frac{EI}{l^{2}}\gamma_{n}^{2},$$

(2) $$\gamma_{n}=n\pi +A\psi_{n}+B\psi_{n},$$

(3) $$\psi_{n}=\sqrt{\frac{EI}{{m{\left(2\pi f_{n}\right)}^{2}l}^{4}}},$$

(4) $$A=-18.9+26.2n+15.1n^{2},$$

(5) $$B=\left\{\begin{array}{c} 290\;(n=1)\\ 0\;(n\geq 2) \end{array}\right.,$$

where T is the cable tension force, m is the mass density per unit length, l is the cable length, n is the vibration mode number, fn is nth natural frequency, and EI is the flexural rigidity of a cable.

Because of the accuracy of Equation (3) found during its assessment using numerical and experimental results [10,30], it was selected as the equation to calculate the tension force in cables.

In some instances, obtaining the current values for the length, mass, and flexural rigidity for all the cables within a bridge can be challenging. However, this could be addressed by conducting a preliminary survey using an alternative measurement methodology, for instance, direct tension determination through a hydraulic jack and the use of an accelerometer to estimate vibration frequency. These parameters can be collectively represented by a constant, C, reflecting the cable characteristics. Substituting this constant in Equation (1) yields:

(6) $$\frac{T_{d}}{f^{2}}=\frac{4ml^{2}}{\gamma_{n}^{2}}-\frac{EI{*\gamma }_{n}^{2}}{l^{2}*f^{2}}=C,$$

where $T_{d}$ is the direct tension force estimation, f is equal to $\frac{fn}{n}$, and C is the constant calculated from the simultaneous measurement of tension and frequencies. Then, by combining Equations (1) and (6), the indirect tension force can be computed as follows:

(7) $$T_{i}=\frac{T_{d}}{{\left(f1\right)}^{2}}*{\left(f2\right)}^{2}=C*{f2}^{2},$$

where $T_{i}$ is the indirect tension measurement, f1 is the vibration frequency measured at the same time as the direct tension, and f2 is the vibration mode frequency posteriorly determined.

Since the indirect tension force is calculated using three independent measurements (direct tension, initial vibration frequency, and subsequent vibration frequency) with independent errors, it is subject to error propagation. The following Equation (8), as provided in [31], can be applied to model the effects of the independent measurements on the tension force estimation:

(8) $$\sigma_{z}=\sqrt{{\left(\frac{\delta_{z}}{\delta_{x1}}*\sigma_{x1}\right)}^{2}+{\left(\frac{\delta_{z}}{\delta_{x2}}*\sigma_{x2}\right)}^{2}+\ldots +{\left(\frac{\delta_{z}}{\delta_{xn}}*\sigma_{xn}\right)}^{2}},$$

where $\sigma_{z}$ is the standard deviation, $\frac{\delta_{z}}{\delta_{x}}$ is the partial derivative of z concerning x (independent measurement), and $\sigma_{x}$ is the standard deviation of the measurement. To analyze the error propagation of this alternative methodology, it is necessary to substitute Equation (7) in (8), which yields:

(9) $$\sigma_{T_{i}}=\sqrt{{\left(\frac{{f2}^{2}}{{f1}^{2}}*\sigma_{T_{d}}\right)}^{2}+{\left(\frac{-2*T_{d}*{f2}^{2}}{{f1}^{3}}*\sigma_{f1}\right)}^{2}+{\left(\frac{2*f2*T_{d}}{{f1}^{2}}*\sigma_{f2}\right)}^{2}},$$

where $\sigma_{T_{i}}$ is the standard deviation of the indirect tension force calculation, $\sigma_{T_{d}}$ is the standard deviation of the direct tension force measurement, and $\sigma_{f1}$ and $\sigma_{f2}$ are the standard deviations of the first and last estimation of vibration frequency, respectively.

**Figure 2:**
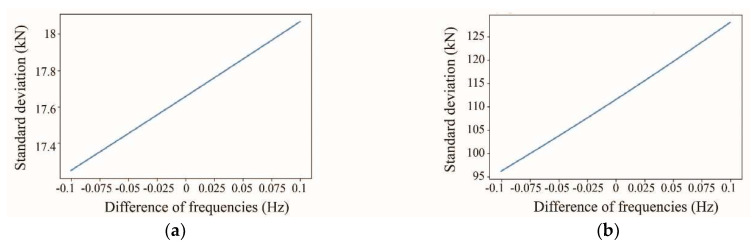
Propagation error for cables: (**a**) on the 1st position, (**b**) on the 14th position.

Analyzing Equation (9), it is observed that the first term (${\left(\frac{{f2}^{2}}{{f1}^{2}}*\sigma_{T_{d}}\right)}^{2}$) provides an error value close to the squared standard deviation of the direct tension. The reason for this is that the first measurement of vibration frequencies is divided by the second one, resulting in a value similar to 1, which means that the resulting error is almost equal to the squared standard deviation of the direct tension. In the case of the second (${\left(\frac{-2*T_{d}*{f2}^{2}}{{f1}^{3}}*\sigma_{f1}\right)}^{2}$) and third terms (${\left(\frac{2*f2*T_{d}}{{f1}^{2}}*\sigma_{f2}\right)}^{2}$), if the values of vibration frequency and their standard deviation are the same, the resulting errors are equal. The last two terms could provide an error value equal to several times the squared standard deviation of the vibration frequencies. The reason for this is that the direct tension value is involved as the numerator of the division against the vibration frequencies. Additionally, it can be established that the higher the value of the constant, C, the higher the error in the terms related to the last frequency measurement, $\sigma_{f2}$. Figure 2a and Figure 2b depict a simulation of error propagation for cables on the 1st and 14th positions of semi-harp number 2, respectively. The simulation compares the standard deviation error in kN with respect to the difference in vibration frequencies between f1 and f2 (f1 is a fixed value and f2 varies around it). The figure shows that cables in the 1st position exhibit higher error tolerance than the longer cables on the bridge. The horizontal axis represents the variation in the last frequency estimation, f2, showing increased propagation error as it deviates. For instance, in the cable at the 1st position (Figure 2a), the lowest standard deviation is 17 kN, with a −0.1 decrement in the last frequency measurement, f2, and the highest standard deviation is 18 kN, with a 0.1 increment in it. In the case of the cable at the 14th position (Figure 2b), the lowest standard deviation is approximately 98 kN, while the highest is 127 kN. For an easier interpretation, the examples were calculated based on historical data, assuming that all the data was collected with an accuracy of ±9.8 kN and ±0.02 Hz for direct tension force and frequency detection, respectively.

The short cables exhibit a low propagation of errors in the estimated tension force, but the standard deviation of the frequency estimation is higher due to their high-frequency range (Table 1). In contrast, the cable in the 14th position has a low standard deviation in the frequency estimation due its low-frequency range. However, the standard deviation of the indirect tension is higher than the one calculated for short cables. Nevertheless, the propagation error can be mitigated by employing an average of constants, C, when multiple simultaneous measurements are taken between direct tension and frequency determination. Additionally, better results can be achieved by using mean values of tension force that consider all the identified natural frequencies in the frequency domain [14].

(10) $$\bar{T}=\sum_{j=1}^{n}\left(4ml^{2}\frac{f_{j}^{2}}{\gamma_{j}^{2}}-\frac{EI}{l^{2}}\gamma_{j}^{2}\right),$$

where $\bar{T}$ is the mean tension force, and $f_{j}$ is the jth natural frequency corresponding to the j vibration mode number.

### 3.2. Percent Difference

After analyzing the propagation error of the indirect tension force, it is necessary to ascertain whether this method is sufficiently accurate for monitoring this parameter in cables supporting a bridge. In this context, the indirect tension and the standard deviation needs to be compared with the maximum tension of the threshold design for the cables. This value represents the service limit state, imposing restrictions on stress when the structure operates under normal performance conditions [32]. In this case, it was calculated based on 45% of the load applied to the cable, ensuring the steel reaches its ultimate stress. Maintaining the tension force of the cables within the design limits is crucial for the regular performance of the cable-stayed bridge.

**Figure 3:**
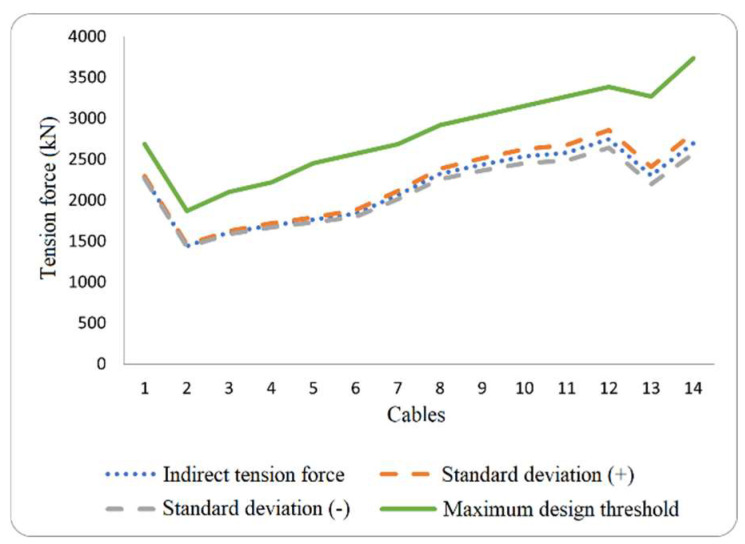
Indirect tension force estimation for cables within the semi-harp number 5 again their maximum design limits.

**Figure 4:**
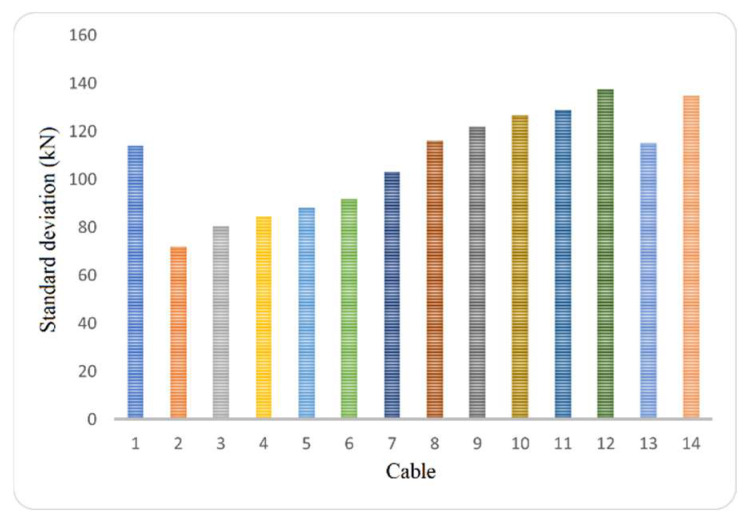
Representation of the standard deviation in percentage (5%) of the total tension force.

If the propagation error range exceeds the maximum design threshold of the cable, it indicates a need for improving measurement accuracy. For all the cables of the Rio Papaloapan Bridge, a standard deviation of 5% of the total tension does not pose a risk to the structure because the tension force is not close to the design threshold (some values are indicated in Figure 3 by the green line). Therefore, during different validation tests, the percent difference must be evaluated based on the reference parameters in each measurement survey, calculated using the following equation:

(11) $$PD=\frac{V_{M}-V_{R}}{V_{R}}*100$$

where PD is the percent difference, $V_{M}$ is measured value, and $V_{R}$ is referenced value. Figure 3 illustrates the tension force values for all the cables in semi-harp number 5, with a standard deviation that covers ±5% of the total tension force. Meanwhile, Figure 4 depicts the relationship between the percentage of total tension (5%) and the standard deviation.

### 3.3. Validation Test for the Proposed Methodology with Accelerometers

In the history of the Rio Papaloapan Bridge, numerous simultaneous measurements between hydraulic jacks and accelerometers have been conducted. Consequently, the proposed methodology could be validated using the historical frequency measurements of each cable. It is acknowledged that the hydraulic jack is not the optimal choice for determining tension force; however, it provided the only available information considered as reference. In this trial, 13 random cables were analyzed, and Table 2 presents the cable positions and semi-harp.

In these cases, the constant, C (Equation (7)), represents an average value calculated using information from 2016 to 2021. The number of elements employed to compute the constant average value varies for each cable due to its historical information.

The validation trial involved calculating the indirect tension force of the 13 cables described in Table 2 using Equation (7). This implies multiplying the constant average value by the new vibration frequency estimation obtained by accelerometers. To obtain the final tension force value, an average was taken using the first five natural frequencies of the cable (Equation (10)). Ultimately, the indirect tension force was compared to the direct tension force determined by the hydraulic jack. The accelerometer used in this test was a G-Link-LXRS from Lord MicroStrain with a measurement range of ±2 g standard, an accelerometer bandwidth from 0 to 500 Hz, an accuracy of 10 mg, and a resolution of 12 bit [33].

**Figure 5:**
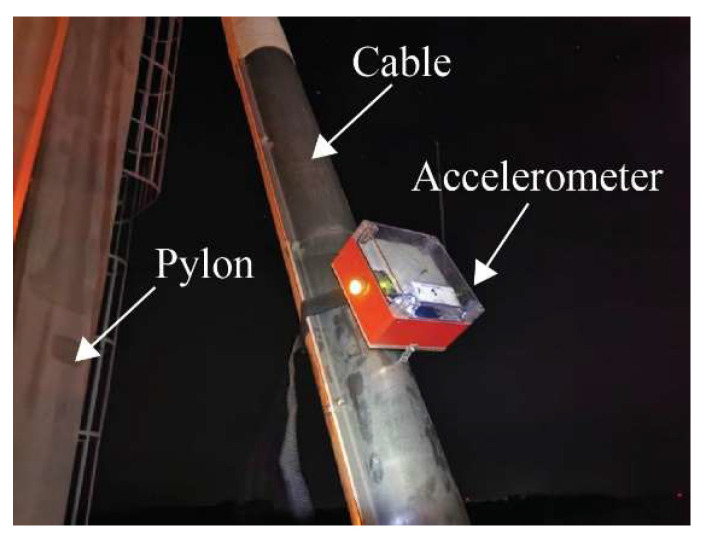
Accelerometer on a cable during the measurement campaign.

The measurements were conducted during the night from 23:00 to 03:00 h, when the bridge was closed for vehicle traffic. Acceleration data was recorded with a sampling frequency of 128 Hz for 2 min (Figure 5). Following that, the frequency of the first five vibration modes of each cable was analyzed using Fast Fourier transform (FFT), followed by the identification of the maximum intensity peaks in the spectrum. Finally, each of the detected frequencies was multiplied by its corresponding constant to calculate the average tension force. Moreover, a hydraulic jack was used to directly measure the tension force at three different times, and the average value was considered as the reference.

**Figure 6:**
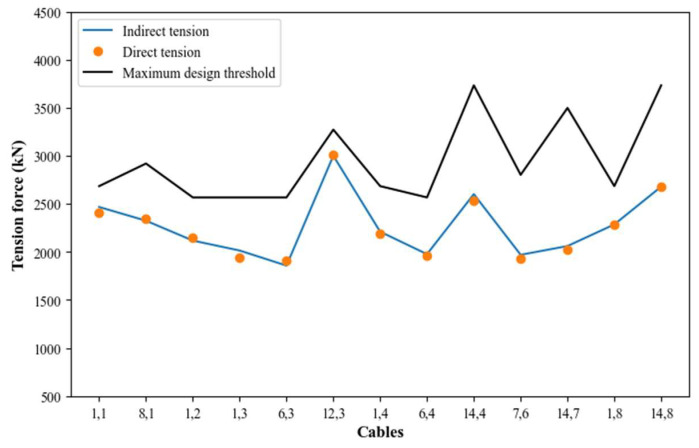
Indirect tension force, direct tension force, and maximum design threshold.

The comparison between direct and indirect measurements is presented in Table 3, where the average percentage difference is 1.48% and the standard deviation is 1.15%. The maximum difference value is 3.8% corresponding to the cable in the 1st position, semi-harp number 3, while the minimum value is 0.13% corresponding to the cable in the 1st position, semi-harp number 8. According to the results, this methodology provides an indirect tension force estimation for the cables within 5% of difference (as previously estimated) concerning the reference methodology used for the constants’ calibration (Figure 6).

According to the reported results of this test, it is defined that the proposed methodology could then be adopted for monitoring the tension force value of the cables from the Rio Papaloapan Bridge.

### 3.4. Computer Vision

The computer vision methodology was employed as an alternative for determining tension force from cables of bridges due to its advantage in remotely recollecting data. This methodology implements the validated process to define tension forces without mechanical parameters of the cable but contemplates a reference value from historical data. It is important to mention that even when no information is available, it can be implemented in three steps: (1) determine the tension of each cable with any direct methodology and simultaneously measure the vibration frequency; (2) estimate the constant value of C; (3) measure the vibration frequency and use the constant C to estimate the tension force. Steps 1 and 2 must be carried out only once; however, if the mechanical parameters of the cables do not change, step 3 can be performed as needed afterward. Sometimes the development of the direct measurements of tension force could be expensive; therefore, it is recommended to be conducted when the bridge is new. In this case, the historical vibration frequency and the tension force of the cables were collected by accelerometers and hydraulic jack, respectively; however, it can be carried out with any measurement methodology able to determine these values accurately.

It is worth noting that Equation (7) can be implemented in several measurement campaigns as long as the cables do not present changes in their physical properties and protection systems (plastic, sheath, elastomeric tapes, and wax). If there are changes in mass per unit length, length, or another parameter, the estimation of the tension force would be inaccurate.

Additionally, the proposed methodology solves the problem when it is not possible to capture video outside of the bridge, capturing information perpendicularly close to the inferior anchor of the cable (the zone of the cable that presents the lowest displacements). In this research, a camera from an iPhone 12 pro (Apple Inc., Cupertino, CA, USA) was implemented, along with the OpenCV library 4.9 in Python 3.11 [34] to process the information. Three trials were developed, the first one in the laboratory environment using a vibrating table, the second one in the Rio Papaloapan Bridge, recording data at the same time with an accelerometer, and the last one was carried out in the structure with the aim of determining the capabilities of the methodology in non-illumination conditions with other temperatures values.

#### 3.4.1. Camera

The camera implemented during this work is part of a mobile phone iPhone 12 pro. It has a focal length of 26 mm, F# of 1.6, sensor size of 56.6 mm × 42.3 mm, and 3840 × 2160 pixels [35].

#### 3.4.2. Digital Image Processing Algorithm

**Figure 7:**
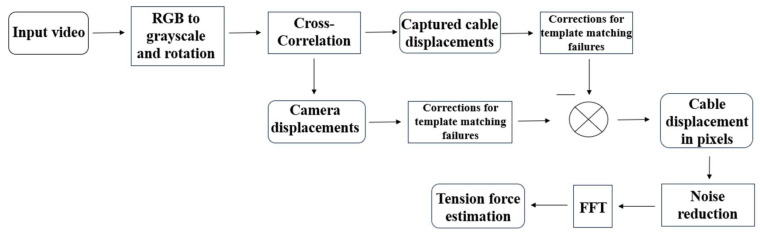
Image-processing algorithm.

The proposed methodology to determine the vibration frequencies of the cables is outlined in Figure 7. First, a calibration step is required to calculate the intrinsic and extrinsic parameters of the camera, compensating for image deformations caused by the camera sensor and its optical elements. Calibration was carried out by taking several pictures of a chess table from different positions. Subsequently, the second step of the algorithm involves converting the image from RGB (red, blue, green) to grayscale and rotating it to its normal position relative to the camera sensor axis, aiming for a horizontal or vertical position of the cable. The Affine Transformation method [36] was employed to rotate the images, considering the design inclination angles of each cable. The third step is to establish two points of interest as templates to determine displacements using cross-correlation. In this case, the methodology implemented was the Normalized cross-correlation, represented as follows [37]:

(12) $$\alpha_{nccorr\left(x,y\right)}=\frac{\sum_{x’,y’}\left(T(x’,y’)*I(x+x’,y+y’)\right)}{\sqrt{\sum_{x’,y’}{\left[T(x’,y’)\right]}^{2}*\sum_{x’,y’}{\left[I(x+x’,y+y’)\right]}^{2}}},$$

where $\alpha_{nccorr\left(x,y\right)}$ is the normalized cross-correlation (values from 0 to 1), I is the intensity from a section of the image in grayscale that is compared against the template, T is the templates image intensity in grayscale, and $\left(x,y\right)-(x’,y’)$ are the coordinates of the searching regions in the images. Through a fast visual inspection, both points are selected. The first one must be a distinctive element within the cable, such as a screw, a wire, or a stain. In the case of the second one, it must be a stable element outside of the bridge and as close as possible to the structure. The element should not present displacements during the recording session, for instance, a tree or mountain. The two chosen templates determine the camera displacement and the captured cable displacements in pixel units, respectively. With the aim of reducing the template-matching failure, the interest section was reduced, i.e., the frame was adjusted considering just a portion that includes the area of interest of the cable and the inmove element out of the bridge. Additionally, the outlier displacements produced by the template matching failures were eliminated and replaced considering linear interpolation. The elimination of information was carried out by implementing a limit of ±3σ. The last step is to subtract the camera and cable displacements and finally apply a high pass filter, such as the Butterworth one, and an FFT algorithm.

The software implemented in Python was installed on a server. The processing algorithm still needs the help of a user to identify a point on the cable and the one that helps to estimate the camera displacements; however, the rest of the process, such as the rotation, change from RGB to grayscale, cross-correlation, corrections, noise reduction, FFT application, peak selection, and the tension force estimation, is automatic.

#### 3.4.3. Displacement Detection

To calculate the tension of the cable indirectly, it is necessary to know the frequency of its vibration modes in the transversal-axis of the cable, which is measured from its displacements over a certain period. In the proposed method, a camera installed on a tripod at the bridge records a video of a cable in movement. The captured displacement, CD, is a sum of the movements produced by the camera and the cable oscillations, as described in Equation (13):

(13) CD=CAMD+CABD,

where CD is the capture displacement, CAMD is the displacements produced by the camera, and CABD is the cable displacement.

The displacements of a bridge are the result of the combination of several factors, being the bridge’s natural movement and the load applied on the bridge (traffic or pedestrians) the primary contributors. The displacement produced by the natural behavior of the bridge depends on its structure; for instance, as the Rio Papaloapan Bridge is cable-stayed, its displacements are relatively large with low frequencies. Meanwhile, the traffic loads induce displacements with high frequencies. In Table 4, the frequencies of some vibration modes are presented.

Additionally, to this range of frequencies, the tripod of the camera also adds movements to the recorded displacements. Then, Equation (13) can be modified as follows:

(14) CD=(ND+ID)+TD+CABD,

where ND is a natural movement, ID is the induce displacements, TD is the movement produced by the tripod, and CABD is the displacement of the cable.

During the determination of the cable displacement, it is necessary to obtain the movement suffered by the cable with respect to an immobile element (i.e., camera displacements). This can be assessed by using cross-correlation, which searches for a template window within an area of interest, bringing the most similar position to the user’s attention. As a result, four displacement arrays were obtained, two for the camera movements (Xc, Yc) and two for the cable displacements (Xd, Yd). The cross-correlation method returns coordinates position in pixels; hence, the four arrays are in the same units. In this case, a transformation of the displacement from pixels to mm is not required, as the frequency of the vibration mode could be obtained based on displacements in pixels.

Considering the errors generated by the displacement calculation from the cross-correlation, it is convenient to update Equation (14) as follows:

(15) $$CD=\left(ND+ID+TD\right)+CABD+ECAB,$$

where ECAB is the error corresponding to the methodology used during the determination of the cable displacement. Then, the displacements of the camera are subtracted to the captured displacement as follows:

(16) $$CD-\left(ND+ID+TD+EB\right)=CABD+ECAB-EB,$$

where EB is the error related to the determination of the camera movement. Subtracting the apparent displacement of the camera from the captured displacement reveals the behavior of the bridge along with a combination of errors due to the computer vision method. Typically, this type of error is associated with the standoff distance of the camera-object, bridge tracking dynamics, estimation of the camera intrinsic parameters, and dimension information [14]. Finally, following [38], it is recommended to subtract the mean value and the trend of each time series. Additionally, in some cases, outlier data could be identified by establishing a threshold of 3σ and then interpolating them.

Along with the video processing, the main practical strategies implemented to minimize the interference from vehicle-induce bridge vibrations during video acquisitions were as follows: (1) attach a weight at the lowest part of the tripod to provide it stability; (2) the height of the tripod was less than 1.5 m, and, in this way, the displacements of the camera and tripod were reduced; (3) the holder of the iPhone was selected to be steady enough to resist the bridge vibration and wind. Furthermore, the iPhone’s technology has the capability to reduce the vibrations that disturb the acquisition of the video.

#### 3.4.4. From Displacements to Tension Force

The next step is to apply an FFT algorithm to the resulting displacements. FFT breaks down the displacement into its spatial frequency components. In this way, the vibration defined by the displacements can be analyzed in the frequency domain. As a result of the measurement errors and the remaining displacements of the bridge, it is normal that some zones of the graphs present noise, and the frequency of the vibration mode varies.

When the frequency of vibration is extracted from the FFT results, the tension force must be calculated according to the methodology validated in Section 3.3. It indicates that the constant (defined a priori) multiplies the frequency value, resulting in tension force (kN). To improve the accuracy of the methodology, Equation (10) is implemented using the first five natural frequencies of the cable.

#### 3.4.5. Camera Measurements

According to the camera characteristics, the minimum detectable displacement at the pixel level was calculated in relation to the camera-to-cable distance and the following equation:

(17) $$hi=\frac{f_{length}*ho}{f_{length}-do},$$

where $f_{length}$ is the focal length, ho is the real object size, and do is the camera-object distance. Using Equation (17), the magnitude of the real displacement can be determined at the image plane. Subsequently, a minimum threshold must be defined. The minimum limit is twice the pixel size, according to the Nyquist–Shannon theorem. The pixel size is calculated considering the resolution of the image and the size of the charge-coupled device as follows:

(18) $$Ps=\frac{CCD\_size}{pixels},$$

where Ps is the size of the pixel, CCD_size is the size of the charge-coupled device, and *pixels* is the number of pixels in the image. In the case of the camera used for this work, the minimum detectable displacement has an approximate ratio of 2.7 × 10^−5^ m/0.20 m. This characterization is used to verify the field of view and spatial resolution of the displacement measurement according to the camera–cable setup before the trails.

A simulation was developed to determine the relation between accuracy, sensitivity and distances from camera to cables. The displacements of cable number 14 were converted from millimeters to pixels considering the resolution of the images in different distances (1, 2, 3, 4, 5, 6, and 7 m).

From the displacements in pixels, values of less than 1 were substituted for a non-displacement sampling. Subsequently, an FFT algorithm was applied to identify the vibration frequencies. These results are presented in Table 5.

For a simplification estimation of the variation according to the camera-to-cable distance, a mean vibration frequency was calculated per each selected distance; then, it was squared and multiplicated by a constant C with the value of 1950. The results are shown in Table 6.

**Figure 8:**
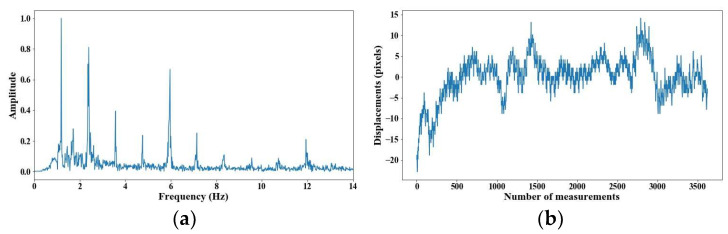
Evaluation of distance, accuracy and resolution (1 m): (**a**) FFT; (**b**) displacements.

**Figure 9:**
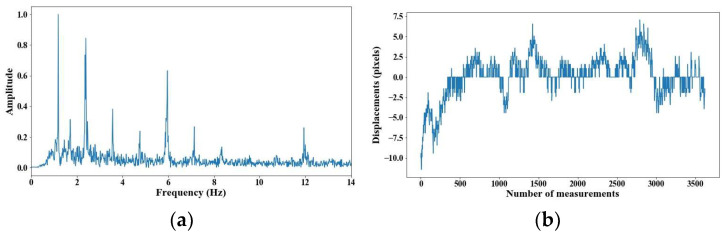
Evaluation of distance, accuracy and resolution (2 m): (**a**) FFT; (**b**) displacements.

**Figure 10:**
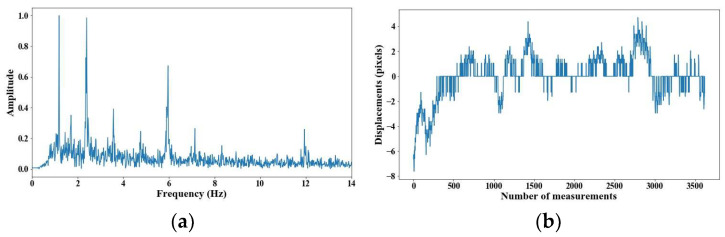
Evaluation of distance, accuracy and resolution (3 m): (**a**) FFT; (**b**) displacements.

**Figure 11:**
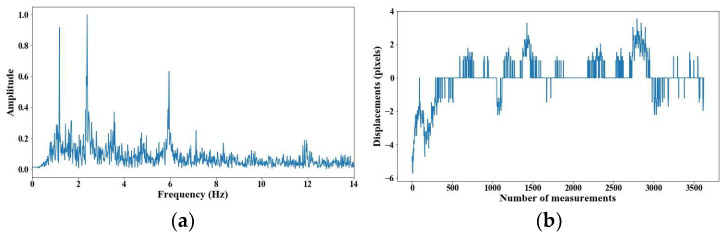
Evaluation of distance, accuracy and resolution (4 m): (**a**) FFT; (**b**) displacements.

**Figure 12:**
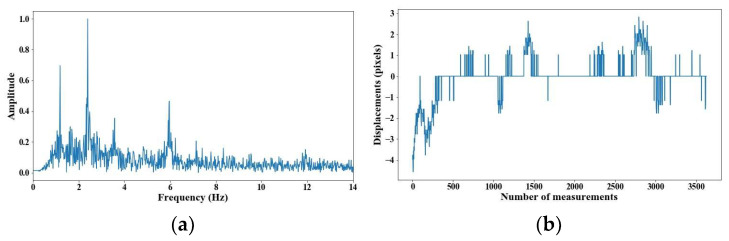
Evaluation of distance, accuracy and resolution (5 m): (**a**) FFT; (**b**) displacements.

**Figure 13:**
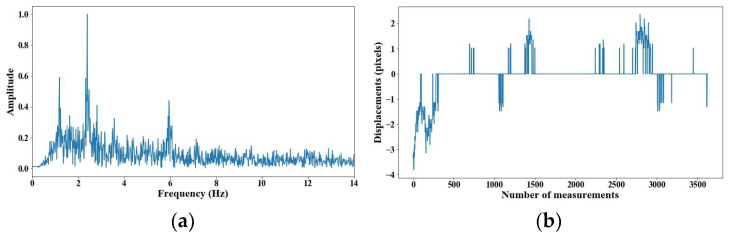
Evaluation of distance, accuracy and resolution (6 m): (**a**) FFT; (**b**) displacements.

**Figure 14:**
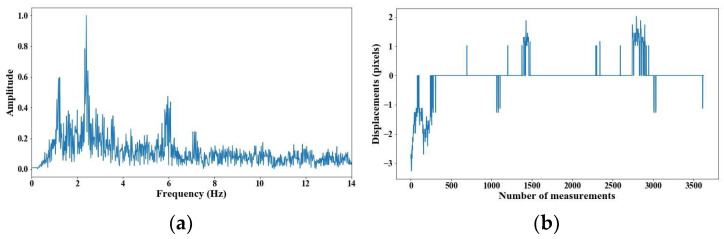
Evaluation of distance, accuracy and resolution (7 m): (**a**) FFT; (**b**) displacements.

From 5 m of distance between the camera and the cable, the tension force was affected; however, up to 7 m of distance, the discrepancies are very notable. From Figure 8, Figure 9, Figure 10, Figure 11, Figure 12, Figure 13 and Figure 14, the results of the frequency and time domains are shown.

According to Table 1 and the Nyquist–Shannon theorem, the sampling frequency required to determine tension force (up to the fifth mode) with video images of all the cables of the Rio Papaloapan Bridge should be 60 frames per second or higher. The cables at the 14th position (longer cables) present the larger displacements; therefore, these have lower frequencies (first mode = 1.16 Hz). In contrast, the cables in the 1st position have the shortest displacements and consequently the highest frequencies (first mode = 7.5 Hz). Using 60 fps allows us to identify up to the fifth mode from the 2nd to 14th cable position, except for the 1st position, where the fifth mode could not be detected.

Likewise, based on the historical tension data of the Rio Papaloapan Bridge, the accuracy for frequency determination has to be ±0.02 Hz or better. Then, considering this value, the resolution in the frequency domain should be lower, which could be achieved by recollecting data for approximately 2 min ($∆f=\frac{1}{\sim 120}\mathrm{Hz}$) or more.

## 4. Experimental Validation

**Figure 15:**
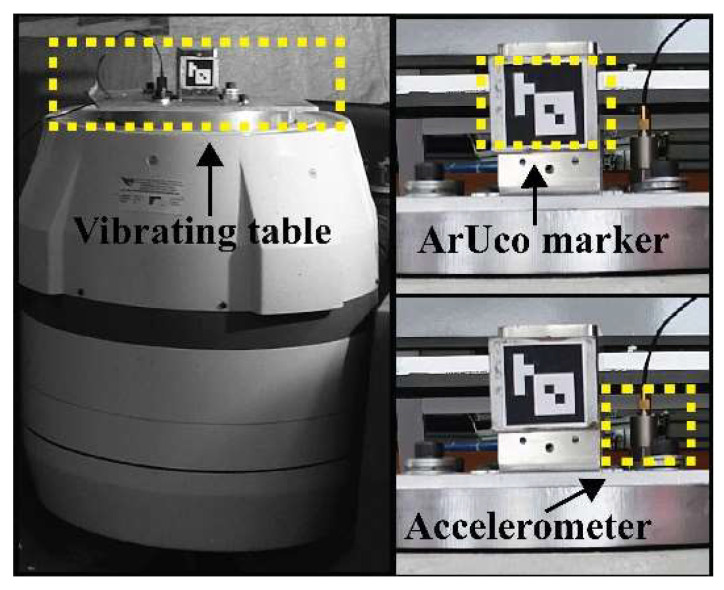
Experimental validation.

The validation trial in the laboratory consisted of placing an ArUco marker of 4 × 4 cm on a vibrating table (Figure 15) and measuring its displacements with a camera, as described in Section 3.4.2. The camera was placed on a tripod installed 70 cm away from the mark to record approximately 2 min of video per session of the table at a sampling rate of 60 fps. The selected sinusoidal displacement frequencies for the vibrating table varied from 26.37 Hz to 11.31 Hz to simulate the average frequency value of the fifth vibration mode of the 2nd–7th cables (Table 1), respectively. With this trial, it is possible to verify that the resolution at the pixel level of the camera is sufficient to correctly capture the cable displacements of the Rio Papaloapan Bridge. Table 7 lists the critical technical specifications used during the laboratory validation phase.

The methodology and the algorithm based on computer vision were assessed considering the vibration frequency of the table as the reference value. Table 8 displays the results of the laboratory testing and the vibrating frequency estimated with the proposed algorithm versus the ground truth set vibration frequency. The Root Mean Square Error (RMSE) was calculated as 0.0022 Hz with a maximum frequency estimation difference of 0.0046 Hz (0.03%) with respect to the set frequency. Additionally, the standard deviation of the repeated sessions (Table 9) was implemented to produce a mean value of them equal to 0.002 Hz. These results confirm that the developed computer vision methodology is viable to determine the vibration frequency of cables as the calculated vibrating frequency drifts less than 0.03% from the original set frequency value of the vibrating table.

## 5. Rio Papaloapan Bridge Trial

The Rio Papaloapan Bridge has a pedestrian zone on both sides, facilitating the safe conduct of non-contact measurement. Two trials were conducted; the first one was under excellent-illumination conditions and a maximum temperature of 33°; meanwhile, the second test was under poor illumination with a maximum temperature of 21°. In this manner, the proposed methodology’s robustness in field-like conditions was rigorously assessed.

**Figure 16:**
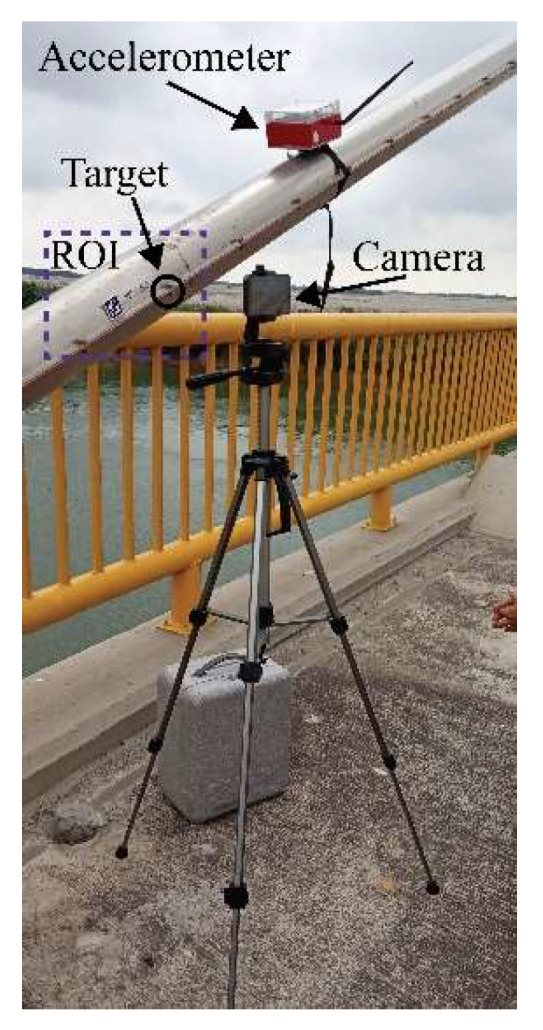
Experimental setup at the Rio Papaloapan Bridge.

The first computer vision trial involved simultaneous measurements of 8 cables of semi-harp number 2 using an accelerometer and camera, as described in Section 3.3 and Section 3.4, respectively. Random loads, including heavy and light vehicles, affected the bridge during the collection of data, leading to significant displacements. The accelerometer, with a sampling frequency of 128 Hz, was secured to the cables using a belt (Figure 16). These readings were used for comparison with the frequency modes calculated using the proposed non-contact method. The camera, mounted on a tripod 70 cm away from the cables, recorded a 2 min video for each cable at a resolution of 3840 × 2160 pixels and 60 fps. Similarly to the laboratory test, the camera was positioned perpendicular as possible to the cables using a digital inclinometer. However, maintaining this position throughout the field measurements was challenging due to the bridge’s exposure to vehicle loads and environmental effects. Thus, these factors influenced the determination of displacements, as described in Equation (16), as part of the term ECAB. The cables studied were on the 14th, 12th, 10th, 8th, 6th, 5th, 4th, and 2nd positions of the semi-harp number 2, providing a representative sampling of the overall cable behavior within the bridge. At the end of the measurement survey, both sets of results were compared in two ways: firstly, by analyzing the frequency of the first five vibration modes, and secondly, through the tension force in kN. This experiment was carried out from 9:00 a.m. to 13:00 p.m., the maximum wind velocity was 7.41 km/h, and the maximum humidity was 86%. During the whole experiment, it was sunny with a clear line of sight between the camera and cable.

**Figure 17:**
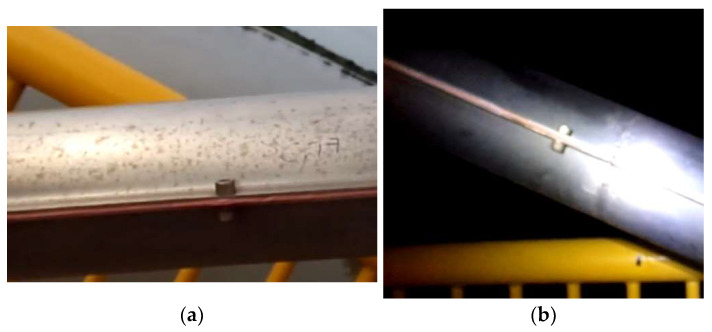
Example of the different conditions during the measurements: (**a**) at day; (**b**) at dawn.

In addition, the second experimental validation was carried out at dawn, that is, under low-lighting conditions, maximum temperature of 21°, maximum wind velocity of 4 km/h, and humidity of 30%. The instrument configuration was the same as in the first test, but the cables evaluated were on 14th position semi-harp 8, 14th position semi-harp 7, 12th position semi-harp 3, 6th position semi-harp 4, 6th position semi-harp 3, 1st position semi-harp 2, and 1st position semi-harp 8. The selected cables represent the hardest, easiest, and most regular cases to evaluate. Moreover, in this trial, the acceleration measurements were performed without vehicle loads, and 5 min before the ones developed by the camera. Figure 17 illustrates the difference between the environmental conditions during the measurements in the Rio Papaloapan Bridge.

The two validation trials were developed considering 15 cables; in Table 10, their main characteristics are described. In general, cables present a specific number of wire strands where each one is formed by seven cables covered with a fully adhesive high-density polyethylene sheath. They are protected from corrosion by three elements: (1) hot-dip galvanizing of the cable; (2) adhesive protective filler; (3) extruded adhesive high-density polyethylene sheath overprotective filler.

## 6. Results

**Figure 18:**
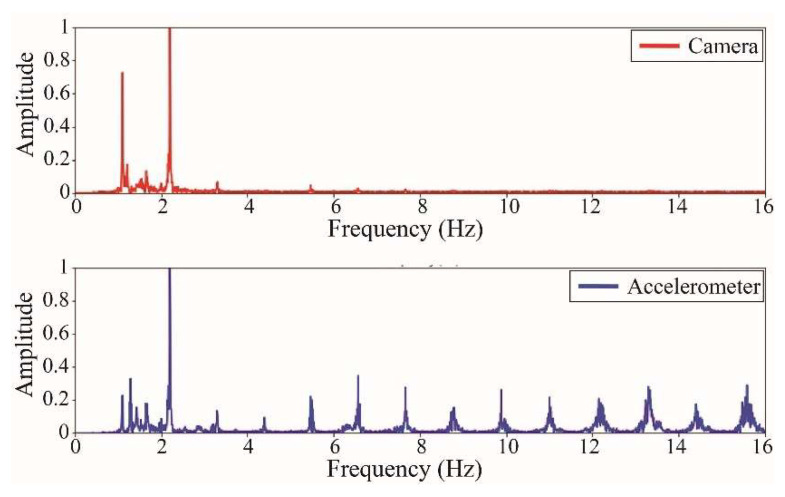
Results of cable on the 14th position in frequency domain.

**Figure 19:**
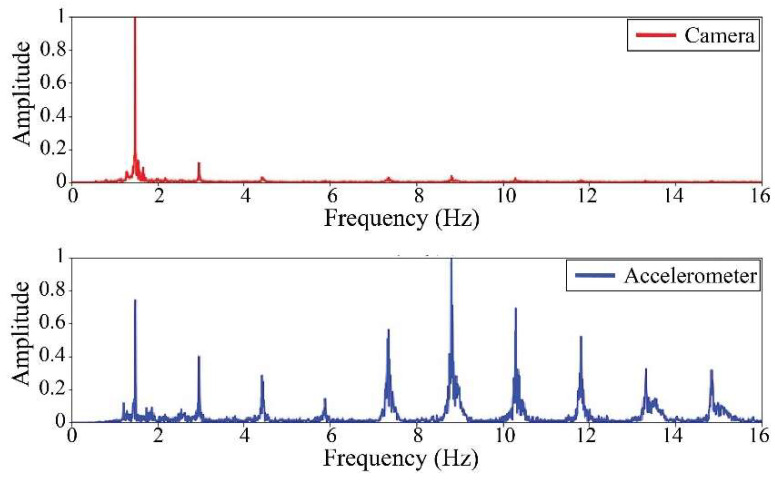
Results of cable on the 12th position in frequency domain.

**Figure 20:**
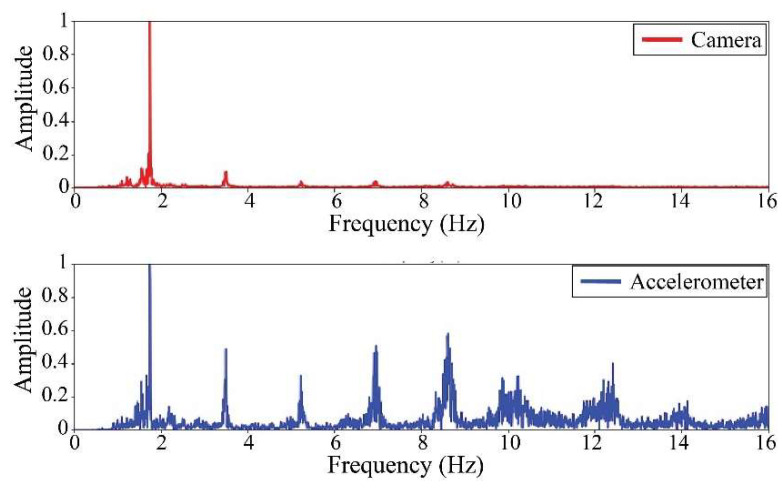
Results of cable on the 10th position in frequency domain.

**Figure 21:**
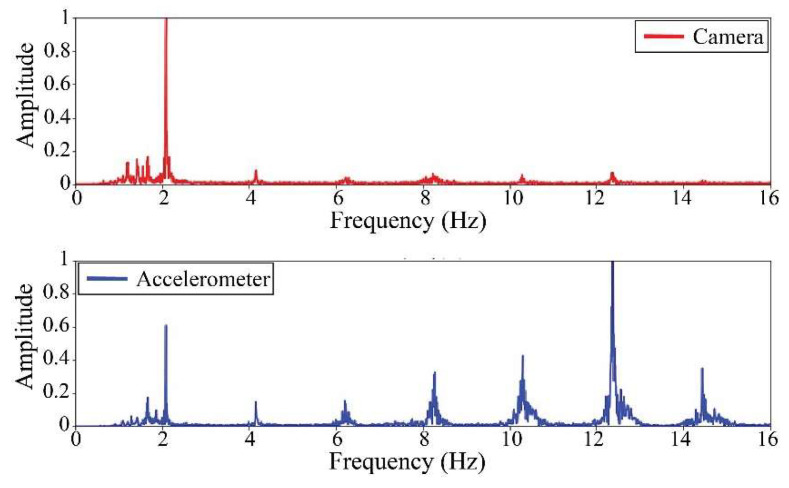
Results of cable on the 8th position in frequency domain.

**Figure 22:**
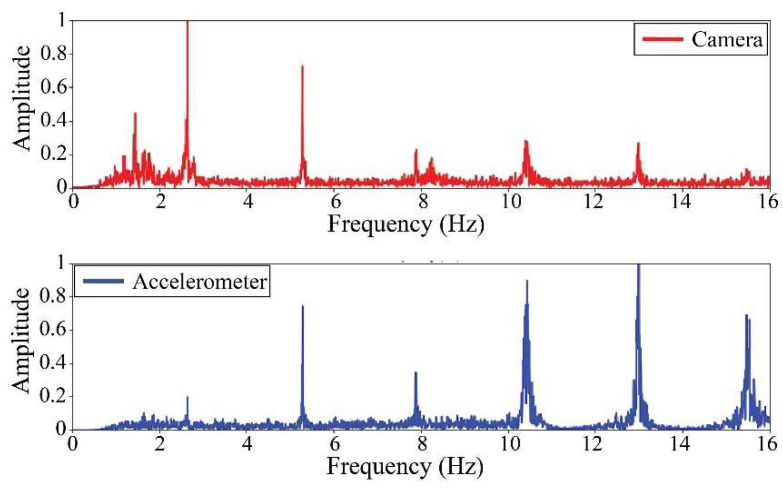
Results of cable on the 6th position in frequency domain.

**Figure 23:**
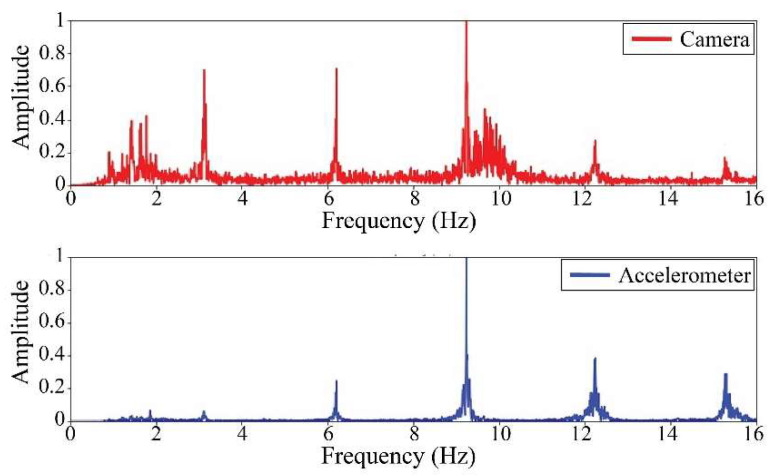
Results of cable on the 5th position in frequency domain.

**Figure 24:**
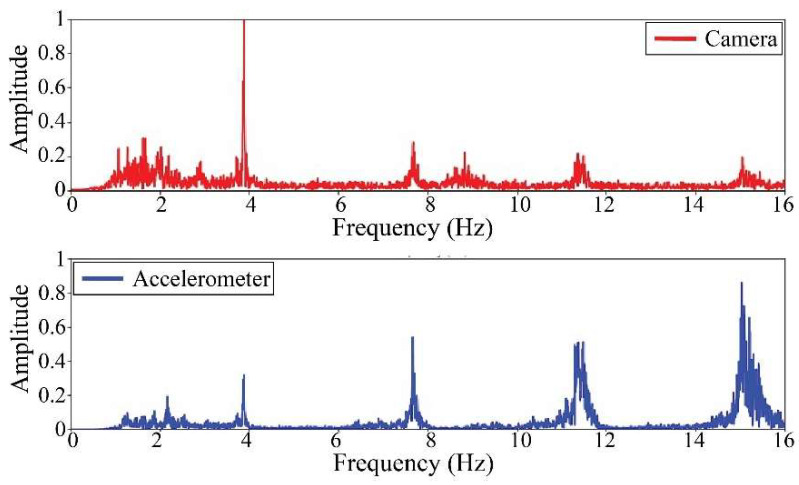
Results of cable on the 4th position in frequency domain.

**Figure 25:**
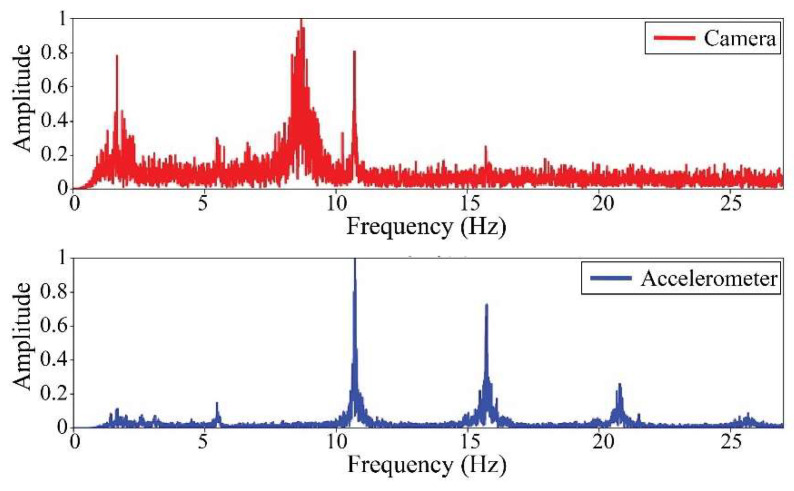
Results of cable on the 2nd position in frequency domain.

The following results were obtained at the Rio Papaloapan Bridge during the first field test described in Section 5. Figure 18, Figure 19, Figure 20, Figure 21, Figure 22, Figure 23, Figure 24 and Figure 25 illustrate the comparison between the camera (red line) and accelerometer (blue line) in the frequency domain with normalized amplitude. The frequency of the different vibration modes was calculated by applying an FFT to the captured oscillations of the cables, as described in Section 3.4.2. In the results shown in the figures, the amplitude of the fundamental frequency determined by the non-contact method for the cables 14th, 12th, 10th, and 8th is too large to distinguish other frequencies. However, this is merely an effect of the graphic scale, and this information can be retrieved by applying a suitable filter in the frequency domain. As for the results of the cables on the 6th, 5th, 4th, and 2nd positions, they exhibit residual noise within a 9 Hz range in the frequency axis. Considering the frequency of the noise, it is likely produced by the movement of the bridge (induced displacements) and from the tripod.

Table 11 presents the sampled frequencies extracted from Figure 18, Figure 19, Figure 20, Figure 21, Figure 22, Figure 23, Figure 24 and Figure 25, and the tension force estimation. Additionally, Table 12 compiles the obtained results, where the largest tension difference was found in cables in the 4th and 2nd positions, with 1.13%, and 2.77%, respectively. This could be attributed to the resolution limit of the camera as the displacements of the cables were smaller than its spatial resolution. Consequently, the only vibration modes captured by the camera were 4 (4th position) and 3 (2nd position). In addition, the lowest difference corresponds to the cable in the 8th position with 0.01%, the mean value of the percentage difference was 0.62%, and the standard deviation equal to 0.94%.

**Figure 26:**
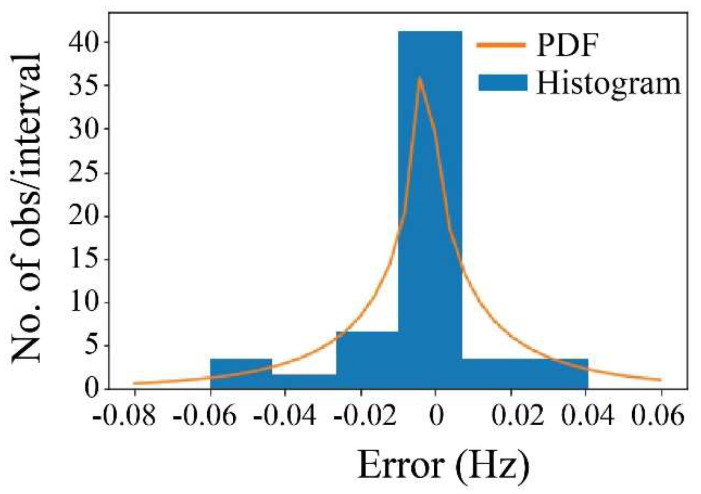
Non-central t-Student distribution.

To analyze the difference in frequency, a statistical distribution was fitted to the histogram of the differences. Considering the Chi-square testing, a suitable function to fit is the non-central t-Student distribution (Figure 26). Therefore, according to the difference’s behavior, it can be established that the probability to obtain a difference in frequency with respect to the accelerometer beyond ±0.02, ±0.04, and ±0.06 was 13%, 6%, and 4%, respectively. This demonstrates that the proposed computer vision methodology has sufficient accuracy to detect the displacement of the bridge’s cables at the pixel levels and relate them to their corresponding tension force.

With the aim of validating the capabilities of the proposed methodology, another measurement campaign was developed. In this case, the tensions were evaluated with an accelerometer that measured without traffic 5 min before. The measurements were carried out in different illumination conditions, being the cables on the 6th position supported by large spotlights; meanwhile, the other cables were brightened by a simple flashlight. The lack of illumination affects the shortest cables on the 1st position; therefore, the results were noisy and unreliable. Considering the rest of the information, the minimum difference was 5.76 kN (0.3%), the maximum was 82 kN (2.7%), the average was 39.29 kN (1.56%), the standard deviation was 31.7 kN (1.17%), and the RMSE was 48.46 kN. Table 13 and Table 14 present the vibration frequencies and the tension force.

**Figure 27:**
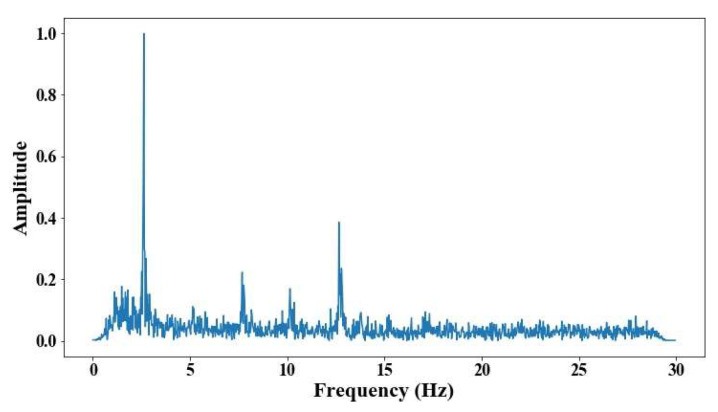
Results of cable on the 6th position semi-harp 4 in frequency domain.

**Figure 28:**
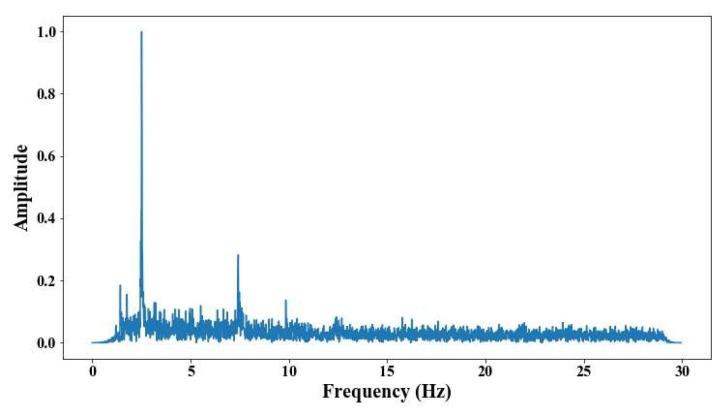
Results of cable on the 6th position semi-harp 3 in frequency domain.

**Figure 29:**
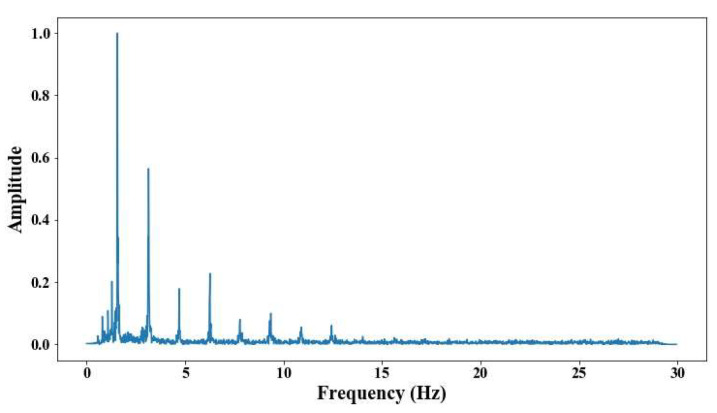
Results of cable on the 12th position semi-harp 3 in frequency domain.

**Figure 30:**
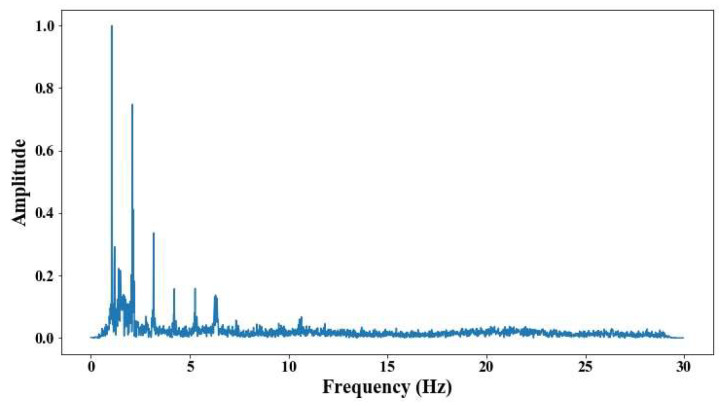
Results of cable on the 14th position semi-harp 7 in frequency domain.

**Figure 31:**
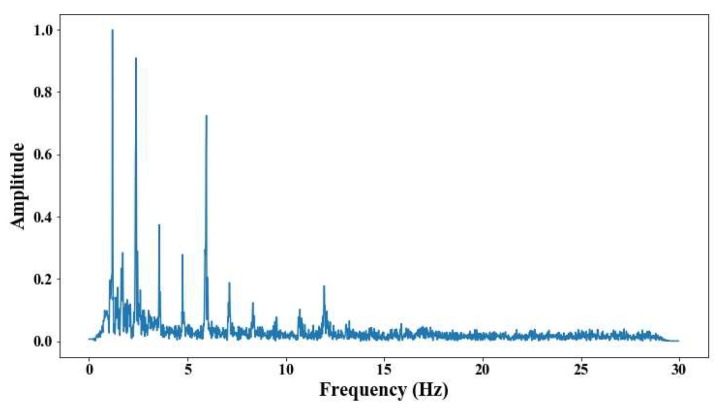
Results of cable on the 14th position semi-harp 8 in frequency domain.

The illumination circumstances changed for each cable; in the case of the elements on the 6th position, the conditions were better. These results indicate the capability of the methodology even with a non-illumination environment; however, the errors in the short cables are even higher than the test developed during the day. Figure 27, Figure 28, Figure 29, Figure 30 and Figure 31 show the results of the measurement under poor-illumination conditions.

In both trials, the reasons for obtaining significant discrepancies were the pixel size of the images and the cable displacement magnitude. The shorter the cable, the lower their displacements and the higher their vibration frequencies; therefore, the frequency domain results are noisy and finding picks leads to less accuracy.

Comparing the trials in the Rio Papaloapan Bridge, it can be claimed that high temperatures could overheat the camera, wind under 7 km/h does not have an effect when the tripod and holder are steady, the angle between the camera and cable can be set up approximately, and it is essential to keep the environment illuminated to detect the cable without any problem.

Along the same lines, there are some strategies to increase accuracy during the evaluation of short cables:

(1) Select a point closer to the middle of the cable, where the displacement of the cable achieves its maximum value.
(2) Carry out the measurements with favorable environmental conditions, excellent illumination, low wind speed, and regular temperature.
(3) Utilize a camera with a higher resolution.

## 7. Conclusions

In this paper, we proposed an alternative methodology for estimating the indirect tension force of cables in stayed-cable bridges without prior knowledge of cross-section area, incorporating historical data of direct tension and vibration frequencies of the cables. The methodology was complemented by a computer vision approach for remote measurements. The overall process involves a short video of the cables with a camera from a certain distance, analyzing the displacements in the Fourier domain to retrieve their vibrating frequency modes.

Validation of the tension force estimation approach was conducted using historical data and comparing its performance against accelerometers and hydraulic jack data from the Rio Papaloapan Bridge. The results showed a difference percentage within 3.8%, indicating that the proposed approach for tension force estimation could be a viable alternative for monitoring the conditions of stayed-cable bridges. It is important to note that the standard deviation of the indirect tension estimation depends on the reference measurement and frequency estimation accuracy.

Regarding the computer vison methodology, it underwent validation in three tests: a laboratory environment and two real-scenario surveys at the Rio Papaloapan Bridge. In the laboratory test, an ArUco marker on a vibrating table generated signals within a frequency range from 11.31 Hz to 26.37 Hz. The camera recorded the marker’s displacement in a 2 min video, resulting in an RMSE of 0.0022 Hz and a mean standard deviation of 0.002 Hz.

In the first field trial at the Rio Papaloapan Bridge, the largest tension differences compared to the accelerometer readings were found in cables at the 4th, and 2nd positions, with 1.13%, and 2.77%, respectively. They could be attributed to the camera detecting fewer vibration modes (4 and 3 vibration modes) for these cables due to smaller displacement magnitude. In contrast, the lowest difference was in the 8th position cable with 0.01%. The overall mean percent difference was 0.62% with a standard deviation of 0.94%. Residual noise within 9 Hz observed in the results of cables on the 6th, 5th, 4th, and 2nd positions could be attributed to induced movement of the camera during the recording period.

The differences between the proposed computer vision methodology and the accelerometers readings were analyzed using a non-central t-Student distribution. The results indicated probabilities of obtaining differences in frequency beyond ±0.02, ±0.04, and ±0.06, equal to 13%, 6%, and 4%, respectively. This demonstrates the sufficient accuracy of the computer vision methodology to detect cable displacements at the pixel level and correlate them with tension force.

On the other hand, the results during the second field trial established that due to the lack of illumination, the frequencies from the short cables are not reliable. Considering the rest of the information, the minimum difference was 5.76 kN (0.3%), the maximum was 82 kN (2.7%), the average was 39.29 kN (1.56%), the standard deviation was 31.7 kN (1.17%) and the RMSE was 48.46 kN. The measurements were carried out in different illumination conditions, being the cables on the 6th position supported by large spotlights; meanwhile, the other cables were brightened by a simple flashlight.

Comparing both trials in the Rio Papaloapan Bridge, it can be claimed that high temperatures could overheat the camera, wind under 7 km/h does not have an effect when the tripod and holder are steady, the angle between the camera and cable can be set up approximately, and it is essential to keep the environment illuminated to detect the cable without any problem.

In conclusion, our proposed indirect method to estimate the tension force of cables in stayed-cable bridges could be used as an alternative inspection method, particularly in scenarios where the mechanical parameters of the cables are unavailable or it is challenging to measure them directly. It is important to mention that other kinds of measurement methodologies for estimating direct tension force and vibration frequency can still be applied. In addition to this, computer vision provides the users with the advantages of cost reduction, ease of operation, and a safer measurement campaign because it is not necessary to access the cables, i.e., data is recorded remotely. In this research, the distance of the camera to the target implemented was around 70 cm. However, depending on the cable characteristics, the results could still be accurate even if the distance is larger. In the case of needing more precision, this method could be improved by incrementing the image resolution or using a subpixel resolution algorithm.

It is relevant to mention that the proposed methodology presented the following limitations:

(1) The cables need to be constantly excited by live loads such as vehicles or wind to obtain accurate results.
(2) The trial was carried out using cables of a bridge with a maximum length of 108 m and a minimum of 18 m. Due to the cable’s physical and mechanical parameters, the short cables presented lower displacements than the camera resolution, which produced low accuracy.
(3) The proposed methodology is intended to be close to the cable; however, an increment in distance would reduce accuracy.
(4) The time to process the whole video with a resolution of 4k could be relatively long.
(5) High temperature affects the camera by overheating it, which turns off the sensor, increasing the time to develop measurements.
(6) The size of the videos is relatively high, affecting the time to download and transmit them.
(7) Illumination clearly affects the results.
(8) The methodology was proposed for specifically monitoring the cables of stayed-cable bridges.

In the future, this methodology can be integrated as part of a permanent Structural Health-Monitoring system to estimate cable tension through high-resolution rotating fixed cameras installed on the bridge towers. The use of cameras would minimize the costs of acquiring and installing accelerometers. Also, cameras could be used for other purposes, such as heavy vehicle classification, initial visual inspection of structural elements, periodical collecting of displacement, and, in combination with other sensors, cameras could even be used to determine vehicle speed and weight.

Finally, in the case of further research, the following statements are planned:

(1) An alternative methodology is proposed to increase the distance between the camera and cables with the purpose of monitoring several cables at the same time.
(2) A new algorithm is proposed to obtain the tension force in real-time with low-cost equipment.

## Figures and Tables

**Table 1:** Average of frequencies in the Rio Papaloapan Bridge.

Cable Position	First Mode (Hz)	Second Mode (Hz)	Third Mode (Hz)	Fourth Mode (Hz)	Fifth Mode (Hz)
1	7.5	14.63	21.71	29.25	36.19
2	5.39	10.57	15.56	20.86	26.37
3	4.54	8.97	13.30	17.59	21.87
4	3.62	7.19	10.69	14.16	17.66
5	2.97	5.91	8.81	11.66	14.53
6	2.58	5.14	7.67	10.17	12.69
7	2.30	4.57	6.83	9.06	11.31
8	2.12	4.22	6.31	8.38	10.43
9	1.90	3.8	5.68	7.55	9.40
10	1.78	3.54	5.30	7.04	8.74
11	1.61	3.21	4.80	6.39	7.96
12	1.51	3.01	4.52	6.01	7.47
13	1.35	2.71	4.06	5.41	6.74
14	1.16	2.32	3.47	4.63	5.78

**Table 2:** Analyzed cables during the validation test.

Cable Position	Semi-Harp
1	1
8	1
1	2
1	3
6	3
12	3
1	4
6	4
14	4
7	6
14	7
1	8
14	8

**Table 3:** Results from the validation trial.

Cable Position	Semi-Harp	Indirect Tension Force (kN)	Direct Tension Force (kN)	Maximum Design Threshold	Absolute Difference (kN)	Percent Difference (%)
1	1	2467.74	2410.8	2685.2	56.94	2.36
8	1	2325.24	2342.2	2920.4	16.96	0.72
1	2	2118.37	2146.2	2567.6	27.83	1.3
1	3	2014.69	1940.4	2567.6	74.29	3.8
6	3	1857.82	1911	2567.6	53.18	2.78
12	3	3001.2	3008.6	3273.2	7.4	0.25
1	4	2210.76	2195.2	2685.2	15.56	0.71
6	4	1977.01	1960	2567.6	17.01	0.87
14	4	2601.6	2538.2	3733.8	63.4	2.5
7	6	1970.3	1930.6	2802.8	39.7	2.06
14	7	2061.52	2028.6	3498.6	32.92	1.62
1	8	2286.47	2283.4	2685.2	3.07	0.13
14	8	2679.48	2675.4	3733.8	4.08	0.15

**Table 4:** Frequency of the first vibration modes of the Rio Papaloapan Bridge.

Vibration Mode	Frequency (Hz)
1	0.4101
2	0.5700
3	0.6471
4	0.7997
5	0.9032
6	0.9925
7	1.0978
8	1.1985
9	1.4149
10	1.6785
11	1.7655
12	1.9794
13	2.5574
14	2.7993

**Table 5:** Frequency of the first vibration modes according to the camera-to-cable distance.

Vibration Mode	Frequencies Found in 1 m of Distance (Hz)	Frequencies Found in 2 m of Distance (Hz)	Frequencies Found in 3 m of Distance (Hz)	Frequencies Found in 4 m of Distance (Hz)	Frequencies Found in 5 m of Distance (Hz)
1	1.1923	1.1923	1.1923	1.192	1.192
2	2.4013	2.4013	2.4013	2.4006	2.4006
3	3.5774	3.5771	3.5771	3.5761	3.5761
4	4.7695	4.7695	4.7695	--	--
5	5.9784	5.9784	5.9784	5.9768	5.9765
6	7.1543	7.1542	7.1542	7.1523	--

**Table 6:** Tension force estimation considering a distance between the camera and cable of 1, 2, 3, 4, 5, 6, and 7 m.

Distance (m)	Tension Force (kN)
1	2781.43
2	2781.33
3	2781.33
4	2781.61
5	2784.22
6	2783.62
7	2809.40

**Table 7:** Critical technical specifications used during the laboratory validation phase.

Technical Specifications	Technical Specifications
Vibration frequency	11.31, 12.69, 14.53, 17.66, 21.87, 26.37 Hz
Acceleration	10 m/s^2^
Distance camera to vibrating table	70 cm
Sampling frequency	60 fps
Resolution	3840 × 2160 pixels
Duration session	2 min

**Table 8:** Experimental results from the laboratory trial.

Test Number	Frequency of the Vibratory Table (Hz)	Acceleration of the Vibratory Test (m/s^2^)	Frequency Probated by the Camera (Hz)	Absolute Difference (Hz)	Percent Difference (%)
1	26.37	10	26.3729	0.0029	0.01
2	26.37	10	26.3726	0.0026	0.009
3	26.37	10	26.3689	0.0011	0.004
4	21.87	10	21.8731	0.0031	0.014
5	21.87	10	21.8716	0.0016	0.007
6	21.87	10	21.8701	1 × 10^−4^	0.0004
7	17.66	10	17.6594	0.0006	0.003
8	17.66	10	17.6605	0.0005	0.002
9	17.66	10	17.6625	0.0025	0.014
10	14.53	10	14.5286	0.0014	0.009
11	14.53	10	14.5268	0.0032	0.022
12	14.53	10	14.5306	0.0006	0.0041
13	12.69	10	12.692	0.002	0.0157
14	12.69	10	12.6854	0.0046	0.0362
15	12.69	10	12.6927	0.0027	0.021
16	11.31	10	11.31	0	0
17	11.31	10	11.308	0.002	0.0176
18	11.31	10	11.3075	0.0025	0.0221

**Table 9:** Standard deviation of the laboratory trial.

Frequency of the Vibratory Table (Hz)	Standard Deviation (Hz)
26.37	0.0022
21.87	0.0015
17.66	0.0016
14.53	0.0019
12.69	0.0040
11.31	0.0013

**Table 10:** Mechanical characteristics of the evaluated cables.

Cable Position	Semi-Harp Number	Number of Wire Strand	Dimensions (m)	Mass (kg/m)	Cable Position
14	2	30	108.09	36	14
12	2	28	93.74	34	12
10	2	27	79.46	33	10
8	2	25	65.26	30	8
6	2	22	51.23	27	6
5	2	21	44.33	25	5
4	2	19	37.55	23	4
2	2	14	24.64	17	2
14	8	32	108.09	39	14
14	7	30	108.09	37	14
12	3	28	93.74	34	12
6	4	22	51.23	27	6
6	3	22	51.23	27	6
1	8	23	18.38	28	1
1	2	22	18.38	27	1
12	2	28	93.74	34	12

**Table 11:** Results from the first Rio Papaloapan Bridge trial.

**Cable on the 14th position Semi-Harp 2**							
**Instruments**	**1 (Hz)**	**2 (Hz)**	**3 (Hz)**	**4 (Hz)**	**5 (Hz)**	**Mean (Hz)**	**Results (kN)**
Camera	1.1066	2.2059	3.3126	4.4629	5.4749	1.1041	2165.1
Accelerometer	1.10635	2.205522	3.31904	4.40297	5.47194	1.10037	2154.2
**Cable on the 12th position Semi-Harp 2**							
**Instruments**	**1 (Hz)**	**2 (Hz)**	**3 (Hz)**	**4 (Hz)**	**5 (Hz)**	**Mean (Hz)**	**Results (kN)**
Camera	1.4869	2.959	4.4239	---	7.3535	1.4748	2806.36
Accelerometer	1.48294	2.9658	4.4339	5.88727	7.35538	1.4750	2804.02
**Cable on the 10th position Semi-Harp 2**							
**Instruments**	**1 (Hz)**	**2 (Hz)**	**3 (Hz)**	**4 (Hz)**	**5 (Hz)**	**Mean (Hz)**	**Results (kN)**
Camera	1.7527	3.4981	5.2214	6.9594	8.6238	1.7370	2649.69
Accelerometer	1.75105	3.49455	5.2305	6.90607	8.60428	1.73243	2639.02
**Cable on the 8th position Semi-Harp 2**							
**Instruments**	**1 (Hz)**	**2 (Hz)**	**3 (Hz)**	**4 (Hz)**	**5 (Hz)**	**Mean (Hz)**	**Results (kN)**
Camera	2.0940	4.1659	6.2305	8.2364	10.2936	2.0680	2328.16
Accelerometer	2.0915	4.1679	6.2215	8.2676	10.2758	2.06829	2328.03
**Cable on the 6th position Semi-Harp 2**							
**Instruments**	**1 (Hz)**	**2 (Hz)**	**3 (Hz)**	**4 (Hz)**	**5 (Hz)**	**Mean (Hz)**	**Results (kN)**
Camera	2.6544	5.2945	7.9130	10.4096	13.0067	2.6185	2029.44
Accelerometer	2.65236	5.2975	7.89204	10.4505	12.9727	2.61767	2028.16
**Cable on the 5th position Semi-Harp 2**							
**Instruments**	**1 (Hz)**	**2 (Hz)**	**3 (Hz)**	**4 (Hz)**	**5 (Hz)**	**Mean (Hz)**	**Results (kN)**
Camera	3.1258	6.2077	9.2384	12.2471	15.2778	3.07312	2010
Accelerometer	3.13029	6.20854	9.24217	12.2312	15.2797	3.07279	2010.69
**Cable on the 4th position Semi-Harp 2**							
**Instruments**	**1 (Hz)**	**2 (Hz)**	**3 (Hz)**	**4 (Hz)**	**5 (Hz)**	**Mean (Hz)**	**Results (kN)**
Camera	3.8909	7.6866	11.3798	15.0656	---	3.8022	2005.04
Accelerometer	3.88985	7.69211	11.3776	15.0631	18.5735	3.77308	1982.56
**Cable on the 2nd position Semi-Harp 2**							
**Instruments**	**1 (Hz)**	**2 (Hz)**	**3 (Hz)**	**4 (Hz)**	**5 (Hz)**	**Mean (Hz)**	**Results (kN)**
Camera	5.4917	10.7268	15.7127	---	---	5.3218	1281.92
Accelerometer	5.49044	10.7236	15.7143	20.7931	25.8352	5.23711	1247.39

**Table 12:** Summary of the results from the first Rio Papaloapan Bridge trial.

Cable Position	Number of Vibration Modes	Frequencies Standard Deviation	Mean Difference (Hz)	Tension Difference (kN)	Percent Difference (%)
14	5	0.0301	0.0037	10.9	0.51
12	4	0.0074	0.0002	2.34	0.08
10	5	0.0288	0.00457	10.67	0.40
8	5	0.0185	0.00029	0.13	0.01
6	5	0.0286	0.00083	1.28	0.06
5	5	0.0085	0.00033	0.69	0.03
4	4	0.0037	0.0291	22.48	1.13
2	3	0.0026	0.0846	34.53	2.77

**Table 13:** Results of the measurement developed at dawn.

**Cable on the 14th position Semi-Harp 8**							
**Instruments**	**1 (Hz)**	**2 (Hz)**	**3 (Hz)**	**4 (Hz)**	**5 (Hz)**	**Mean (Hz)**	**Results (kN)**
Camera	1.1894	2.3953	3.5682	4.7576	5.9635	1.4199	2649.97
Accelerometer	1.1969	2.4021	3.6156	4.7543	5.9927	1.1974	2679.48
**Cable on the 14th position Semi-Harp 7**							
**Instruments**	**1 (Hz)**	**2 (Hz)**	**3 (Hz)**	**4 (Hz)**	**5 (Hz)**	**Mean (Hz)**	**Results (kN)**
Camera	1.0658	2.1017	3.1676	4.2185	5.2693	1.0548	1999.77
Accelerometer	1.0722	2.1527	3.2	4.2971	5.3278	1.0699	2061.52
**Cable on the 12th position Semi-Harp 3**							
**Instruments**	**1 (Hz)**	**2 (Hz)**	**3 (Hz)**	**4 (Hz)**	**5 (Hz)**	**Mean (Hz)**	**Results (kN)**
Camera	1.5653	3.141	4.6959	6.2612	7.7725	1.5624	3083.2
Accelerometer	1.5377	3.1086	4.6296	6.1423	7.7216	1.5426	3001.2
**Cable on the 6th position Semi-Harp 4**							
**Instruments**	**1 (Hz)**	**2 (Hz)**	**3 (Hz)**	**4 (Hz)**	**5 (Hz)**	**Mean (Hz)**	**Results (kN)**
Camera	2.6204	5.135	7.676	10.1377	12.6523	2.5481	1959.53
Accelerometer	2.6099	5.1616	7.7216	10.1735	12.7086	2.5583	1977.01
**Cable on the 6th position Semi-Harp 3**							
**Instruments**	**1 (Hz)**	**2 (Hz)**	**3 (Hz)**	**4 (Hz)**	**5 (Hz)**	**Mean (Hz)**	**Results (kN)**
Camera	2.5079	--	7.4266	9.8374	--	2.4714	1852.06
Accelerometer	2.5101	4.9704	7.4389	9.8577	12.3512	2.4752	1857.82

**Table 14:** Summary of the results found during the measurements at dawn.

Cable Position	Number of Semi-Harps	Number of Vibration Modes	Tension Difference (kN)	Percent Difference (%)
14	8	5	29.51	1.1
14	7	5	61.7	2.9
12	3	5	82	2.7
6	4	5	17.48	0.8
6	3	3	5.76	0.3

## Data Availability

The original data is not publicly accessible, as it is owned by the bridge’s proprietor. The authors are permitted to use the data entirely for scientific research related to bridge monitoring. However, the complete dataset should not be disclosed.
